# Comparison of Herbarium Label Data and Published Medicinal Use: Herbaria as an Underutilized Source of Ethnobotanical Information

**DOI:** 10.1007/s12231-017-9367-1

**Published:** 2017-03-06

**Authors:** E. N. F. Souza, J. A. Hawkins

**Affiliations:** School of Biological Sciences, University of Reading, Whiteknights, Reading, Berkshire RG6 6BX UK

**Keywords:** Herbaria, ethnobotany, meta-analyses, mode of application, therapeutic use

## Abstract

The use of herbarium specimens as vouchers to support ethnobotanical surveys is well established. However, herbaria may be underutilized resources for ethnobotanical research that depends on the analysis of large datasets compiled across multiple sites. Here, we compare two medicinal use datasets, one sourced from published papers and the other from online herbaria to determine whether herbarium and published data are comparable and to what extent herbarium specimens add new data and fill gaps in our knowledge of geographical extent of plant use. Using Brazilian legumes as a case study, we compiled 1400 use reports from 105 publications and 15 Brazilian herbaria. Of the 319 species in 107 genera with cited medicinal uses, 165 (51%) were recorded only in the literature and 55 (17%) only on herbarium labels. Mode of application, plant part used, or therapeutic use was less often documented by herbarium specimen labels (17% with information) than publications (70%). However, medicinal use of 21 of the 128 species known from only one report in the literature was substantiated from independently collected herbarium specimens, and 58 new therapeutic applications, 25 new plant parts, and 16 new modes of application were added for species known from the literature. Thus, when literature reports are few or information-poor, herbarium data can both validate and augment these reports. Herbarium data can also provide insights into the history and geographical extent of use that are not captured in publications.

**Electronic supplementary material:**

The online version of this article (doi:10.1007/s12231-017-9367-1) contains supplementary material, which is available to authorized users.

## Introduction

Ethnobotanical research is crucial to understanding relationships between people and their biological environment (Thomas 2003). Global or regional studies which compile and analyze data from multiple literature sources can lead to a general understanding about plant use (de la Torre et al. 2012; de Medeiros et al. 2013; Saslis-Lagoudakis et al. 2012; Weckerle et al. 2011), but the availability of appropriate data is a limitation to broad-scale research (Albuquerque and de Medeiros 2012). Herbaria are repositories of information in the form of vouchers, originally serving economic botany, and increasingly seen primarily as resources for plant taxonomy (Bebber et al. 2010; Van Andel et al. 2012). Today, the wider value of herbaria is appreciated (Lavoie et al. 2013); herbaria worldwide house more than 300 million specimens collected over 400 years and as such are a rich repository of specimen collection dates and localities (Thiers 2014). Nevertheless, the extent to which herbarium specimens may contribute ethnobotanical data not captured in publications, filling gaps in our knowledge and providing data for analyses, remains only partially explored.

Ethnobotanists frequently and routinely collect and cite herbarium specimens; indeed, journals such as *Economic Botany* require voucher specimens to be cited alongside use reports. However, specimens which were not collected as vouchers to support published studies may include use data. The most complete survey to assess the frequency of ethnobotanical information in herbarium collections was that of von Reis (1962, 1968). She reported almost 6800 specimens citing medicinal uses among the 2,500,000 specimens of Harvard Herbarium, despite excluding those published or likely to be known already. Since von Reis enumerated the advantages of searching herbaria for novel reports of use, herbarium surveys have become a minor but established source of ethnobotanical data (Bedigian 2004; de la Torre et al. 2012; Fantz 1991; Jenks and Kim 2013; Krishna et al. 2014; Lampe 1986; Lira and Caballero 2002; Lukhoba et al. 2006; McKenna et al. 2011; Prakash 2011; Van Andel et al. 2014; Vickery 1990; Shinde and Prakash 2015). Since herbaria are rich in historical data, they have found particular use in documenting change (Nesbitt 2014). For example, studies of historic herbaria have used the annotation on specimens to reveal changes in local names and uses (Van Andel et al. 2012—Hermann herbarium) or the changing species composition of pharmacopeia (Birch 2009—Gideon Lincecum Herbarium; De Natale and Pollio 2012—Trotter collection).

The Leguminosae (legumes) is one of the largest plant families (Lewis et al. 2005), overutilized for medicine in some regions (Korea and Ecuador) and underutilized in others (North America), and with many documented uses (Moerman et al. 1999). In Brazil, the family comprises c. 2800 species in more than 200 genera (Brazilian Flora 2020 in construction). The legumes of Brazil are distributed among the six biomes described for Brazil, Amazon Forest (1147 sp.), Atlantic Rainforest (997 sp.), Caatinga (620 sp.), Cerrado (1237 sp.), Pampas (138 sp.), and Pantanal (161 sp.). Ethnobotanical work in Brazil is increasing, particularly in the northeast and southeast regions of Brazil, where there are active research groups. However, in the Amazon, Cerrado, Pantanal, and Pampas, there is a relative deficit of ethnobotanical data in the literature (Ritter et al. 2015).

The complementarity of herbarium and published data on medicinal use is yet to be formally assessed, and the proportion of herbarium specimens with medicinal use information is uncertain, since the von Reis study did not record the number of excluded specimens (Von Reis 1962; Von Altschul 1968; Nesbitt 2014). Here, we compare literature and herbaria as sources of ethnobotanical data, taking medicinal use of Brazilian legumes as a case study. We ask how often herbaria provide data on plant use that is not present in the literature, whether the data provided in the two sources is broadly comparable, and if so, whether the data from herbarium specimens could be used to validate or augment poorly substantiated literature records. By addressing these questions, we seek to outline a role for herbarium data in ethnobotanical research.

## Methods

### Data Collection

Published medicinal use data and data from Brazilian herbarium specimens were compiled for the legumes of Brazil. Data were organized in a database with the following fields: genus and species as recorded in the publication or in the specimen database, currently accepted name, therapeutic application as recorded in the publication or in the specimen database, therapeutic application categorized according to WHO International Classification of Diseases 10 (World Health Organization 2016), parts used, mode of application, and locality. Where necessary, Google maps and the Brazilian Geographic and Statistical Institute online data (IBGE) were used to get geographical coordinates for the localities cited on papers but with no cited coordinates. Generic and species names followed The Plant List (The Plant List 2013) and Missouri Botanical Garden’s Tropicos database (Tropicos.org 2016) and were corrected (standardized) using the Plantminer R script (Carvalho et al. 2010).

Publications citing medicinal uses of legume species in Brazil were identified using Google Scholar and searches of the following journals: Acta Botanica Brasilica, *Economic Botany*, Fitoterapia, Flovet, Journal of Ethnobiology, Journal of Ethnobiology and Ethnomedicine, Journal of Ethnopharmacology, Journal of Medicinal Plants Research, Revista Brasileira de Biociencias, Revista Brasileira de Farmacognosia, and Revista Brasileira de Plantas Medicinais and Rodriguesia. Search terms, which were used both in English and Portuguese, are presented in Table 1. From the subset of papers found which record medicinal plant use, a dataset was compiled to record fields as above.

**Table 1:** Combinations of words and terms used as search terms in the literature review.

Column 1	Column 2
Brazilian ethnobotany^a^	Cerrado
Ethnobotany	Atlantic rainforest
Ethnopharmacology	Caatinga
Medicinal plants	Pantanal/wetlands
Medicinal flora	Pampas/grasslands
Ethnobiology	Amazon forest

Each word or term in column 1 was combined with each in column 2.

^a^The term “Brazilian Ethnobotany” was also searched alone.

Herbarium data were extracted from the online list of herbarium and biological collections from Brazil, Species Link (http://splink.cria.org.br/). The file was exported to excel format, and a search using the keywords “medicinal” and “uses” (in Portuguese) was conducted in order to filter only the specimens with medicinal information. Specimens were sourced from 15 herbaria: HEMACT, INPA, IPA, JBRJ, MBML, UEL, UEM, UEM, UFERSA, UFG, UFMS, UFPB, UFPE, UFRPE, UNESPSJRP, UNICAMP.

### Data Analysis

The data from the surveys were explored in several ways to draw out, for the different sources, how much data there were, whether they were comparable or complementary, and their spatial and temporal characteristics.

#### Number of Reports and Presence/Absence of Ethnobotanical Data

The numbers of use reports from the literature and from the herbarium were calculated, where a use report is the accepted species name plus all the associated data originating from one publication or one voucher specimen (i.e., entries or rows in Appendix 1). How frequently use reports, whether from publications or herbarium labels, include data on therapeutic application, plant part used, or mode of application was also calculated as percentages.

#### Number of Species Described and Comparison of Species Lists

The number of species reported from literature and from the herbarium sources was determined. By comparing species lists, species unique to either literature or herbarium sources were identified, and their proportions calculated based on the total number of species for each source. Similarly, the species with associated therapeutic indications/modes of application/plant part data were compared between the literature lists and the herbarium lists, and the proportions of species with these data were also calculated. To account for the possibility that the same study contributed both the herbarium and publication data, we scored the number of times authors and collectors were the same person, for records of the same content.

#### Comparison of Information Content

To test whether the information content of the data from the two sources was similar, we compared numbers of reports by genus, for each therapeutic application and in total. To compare therapeutic applications, we classified literature and herbarium reports following the WHO ICD-10 (http://www.who.int/classifications/icd/en/). Total numbers of each therapeutic application, plant part used, and mode of application were recorded for both sources, so that the broad comparability of the data content could be assessed visually and using Spearman rank-order correlation tests.

#### Spatial and Temporal Distribution of Data

The number of reports and species, from both literature and herbaria, was calculated for each biome in Brazil. Their coordinates were plotted to compare spatial distribution of studies and collections. To investigate whether the number of reports in each biome from each source was correlated, Spearman rank-order correlation was used. Dates of collection and publication between the two sources were also compared, and the changing proportions of specimens through time which report any information about therapeutic application, plant part used, or mode of application were assessed.

#### Validation and Augmentation of Datasets

We searched for the species only reported once in the literature (whether data-rich, i.e., including mode of use, plant part, or therapeutic application, or not) in the herbarium reports. We considered the literature report “validated” if there was an independently collected herbarium report of medicinal use for that species (not a voucher specimen collected by the authors of the study). We also listed the species that were reported as medicinal in the literature, but where there were no literature reports of the mode of use, plant part, or therapeutic application, and searched for these in the herbarium data. For these species, we identified the number of herbarium reports of mode of use, plant part used, or therapeutic application that were not in the literature; these reports we considered to augment the literature reports. Finally, considering only the species with reports from both literature and herbarium, we counted the total number of new use reports from each source.

## Results

### Number of Reports and Presence or Absence of Associated Data Describing Use

Considering the data sourced from publications, excluding reports where identification was only to genus level and uses that were not medicinal, there were 938 reports from 104 publications. Of these, a subset of 69% record therapeutic application, 49% provide information about mode of application, and 61% provided information about plant part used (Table 2; Supplementary material). Considering the herbarium data, of the 240,000 specimens from 15 Brazilian herbaria searched, 462 (0.2%) indicated whether the plant was used medicinally. These represented 154 species in 62 genera. Of these specimens, 16% described therapeutic application, 4% provided information about mode of application, and 6% provided information about plant part used (Table 2; Supplementary material).

**Table 2:** Summary of data from literature and from herbarium specimens.

	Literature	Herbaria
Number of use reports	938	462
Number of reports with therapeutic indications	654	76
Number of reports with modes of application	462	19
Number of reports with plant parts used	579	30
Number of reports without further information	284	385
Number of species (number/percentage of unique species)	264 (165/62%)	154 (55/36%)
Number of species with therapeutic indications (number/percentage of unique species)	203 (167/82%)	48 (12/25%)
Number of species with modes of application ((number/percentage of unique species)	162 (149/92%)	16 (3/19%)
Number of species with plant parts used (number/percentage of unique species)	184 (165/90%)	22 (3/14%)
Number of species without further information (number/percentage of unique species)	146 (78/33%)	141 (73/52%)

The number of reports from literature or herbarium sources is indicated and whether they include any further information about use (therapeutic indication, mode of application, plant parts). Species data is also presented. The total number of species with recorded medicinal use is given, also the numbers and proportions of species that are either unique to literature or unique to herbarium (those included in one dataset but not the other). Species with uses are those where use for a species is described in that source; unique species are species for which information about that use (therapeutic indication, mode of application, plant parts) is derived only from that source.

### Number of Species Described and Comparison of Species Lists

In total, 264 species in 97 genera were reported as medicinal in the literature, of which 165 species (62%) were unique from literature. Of the 154 species in 62 genera known from the herbarium, 55 (36%) were known only from that source. The percentages of unique species from literature where therapeutic applications, plant parts used, and modes of application were known were much higher, from 82 to 92%. Nevertheless, in the fewer cases from herbaria, we found 14% of unique species with indicated plant parts used and 25% of unique species with therapeutic indications (Table 2).

### Comparison of Information Content

For publications, descriptions of use could be translated to 16 of the 19 WHO ICD-10 categories of use. For herbaria, therapeutic uses were allocated to 13 of the 19 WHO ICD-10 categories. Herbaria and literature reports showed a statistically significant association between the sets of ranks, when the most cited genera (*p* = <0.0001, rho = 0.88), therapeutic applications (*p* = 1.901e-07, rho = 0.91), modes of application (*p* = 0.002, rho 0.86), and plant parts (*p* = 0.0003, rho = 0.94) were ranked and compared (Tables 3 and 4). The number of records which had the same information and for which collector and author were the same person was 35 (3.6% of records).

**Table 3:** Most cited genera in literature and herbarium.

Literature		Herbarium	
Genus	Count	Genus	Count
*Senna*	116	*Bauhinia*	77
*Bauhinia*	97	*Senna*	64
*Hymenaea*	66	*Mimosa*	27
*Caesalpinia*	65	*Caesalpinia*	23
*Mimosa*	50	*Hymenaea*	16
*Anadenanthera*	34	*Stryphnodendron*	15
*Stryphnodendron*	30	*Copaifera*	14
*Copaifera*	29	*Anadenanthera*	11
*Amburana*	26	*Amburana*	8
*Bowdichia*	23	*Bowdichia*	5
*Desmodium*	14	*Desmodium*	9
*Libidibia*	1	*Libidibia*	9

Counts represent the total number of reports for each genus, in the literature and in the herbarium.

**Table 4:** Comparison of uses between literature and herbaria, showing the number of reports per therapeutic application, plant part used, and mode of application.

Use	Literature reports	Herbarium reports
Therapeutic application		
Diseases of the respiratory system	193	21
Diseases of the digestive system	156	10
Diseases of the blood and blood-forming organs and certain disorders involving the immune mechanism	151	13
Endocrine, nutritional, and metabolic diseases	151	13
Diseases of the genitourinary system	64	4
Diseases of the femalegenito system	62	7
Diseases of the musculoskeletal system and connective tissue	56	5
Diseases of the circulatory system	48	5
Diseases of the nervous system	43	3
Certain infectious and parasitic diseases	41	5
Diseases of the skin and subcutaneous tissue	24	5
Neoplasms	14	1
Diseases of the eye and adnexa	6	0
Diseases of the ear and mastoid process	5	0
Pregnancy, childbirth, and the puerperium	4	0
Others	2	0
Mental and behavioral disorders	0	0
Plant part used		
Bark	288	14
Leaves	199	7
Root	74	12
Fruit	44	1
Seeds	38	1
Flowers	29	0
Whole plant	16	0
Stem	8	0
Mode of application		
Decoction	153	3
Tea	131	11
Infusion	113	2
Syrup	50	2
Alcoholic infusion	44	0
Bath	32	2
Powder	3	0
Oil	3	0
Wine	2	0

### Spatial and Temporal Distribution of Data

**Fig. 1:**
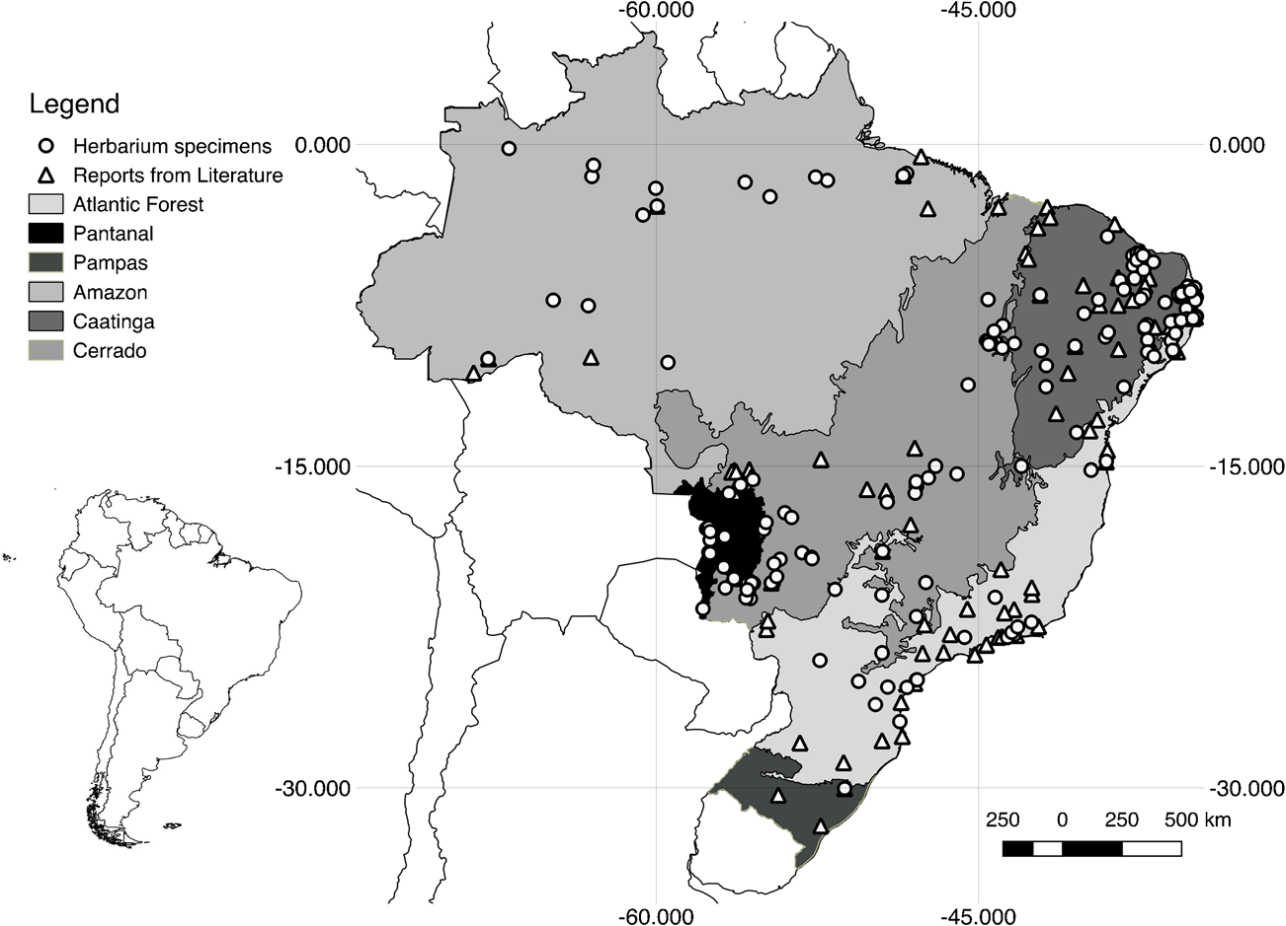
Geographic origin of data extracted from papers (*triangles*) and herbaria (*circles*). *Dots* show the location of studied sites and can represent more than one record.

Figure 1 shows the spatial distribution of data. Considering the geographic origin of literature and herbarium reports combined, the Cerrado and Caatinga biomes were best represented, with 418 and 361 reports, respectively, and the Pampas the most underrepresented, with 12 reports (Table 5).

**Table 5:** Spatial comparison of data from literature and the herbaria.

Biomes	Herbarium		Literature		Total	
	Reports	Species	Reports	Species	Reports	Species
Amazon forest	32 (39%)	22 (39%)	50 (60%)	39 (68%)	82	57
Atlantic forest	74 (43%)	51 (56%)	96 (56%)	56 (61%)	170	91
Caatinga	159 (44%)	54 (55%)	202 (56%)	80 (82%)	361	97
Cerrado	96 (22%)	49 (34%)	322 (77%)	126 (88%)	418	142
Pampas	2 (16%)	2 (18%)	10 (83%)	8 (72%)	12	11
Pantanal	32 (58%)	22 (62%)	23 (41%)	18 (51%)	55	35

The distribution of reports and species from each source in the six Brazilian biomes is presented. The percentages of total reports and total species that each source contributes to each biome are shown.

**Fig. 2:**
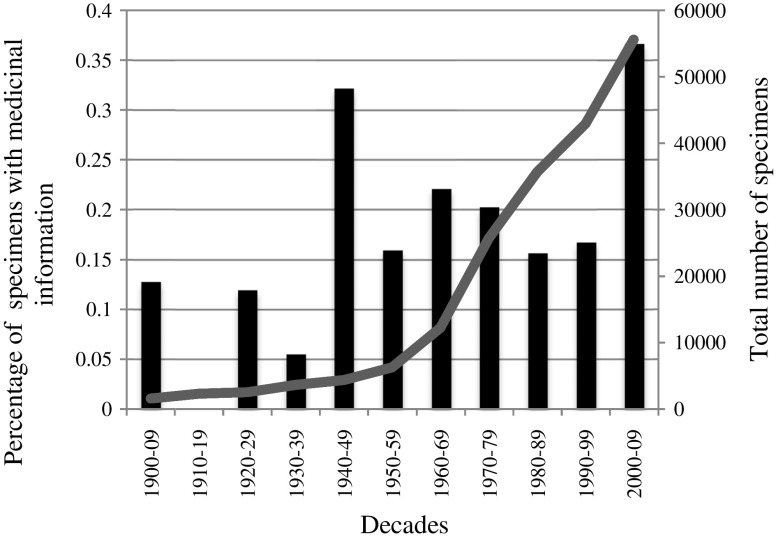
Temporal patterns in the recording of medicinal data in herbaria. The graph shows the total number of herbarium specimens collected in each period and now deposited in the herbaria surveyed (*line*) and the proportion of those specimens with any associated information indicating whether or how the plant is used medicinal (*bars*).

Biomes with more herbarium records also had more literature records (*p* = 0.007, rho = 0.92). Dates of collection and publication comparison revealed herbaria as a unique source of ethnobotanical data until 1980, and the 2000s as the decade with most reports (Table 6). The proportion of herbarium reports with medicinal information increased through time (0.12–0.36% from 1900–1910 to 2000–2009) (Fig. 2). Data for 2010 to 2014 are not plotted: In this period, of 19910 specimens, 23 (0.11%) included information about the medicinal use of the specimen.

**Table 6:** Temporal comparison of data from literature and the herbaria.

Time period	Literature reports	Herbarium reports
1900–1909	0	2
1910–1919	0	0
1920–1929	0	3
1930–1939	0	2
1940–1949	0	14
1950–1959	0	7
1960–1969	0	27
1970–1979	0	51
1980–1989	3	54
1990–1999	20	70
2000–2009	591	203
2010–2014	324	23

The total number of reports in each decade, and in the last 4 years, is reported.

### Validation and Augmentation of Datasets

From the literature, 123 species were reported once (hereafter “singletons”), excluding 5 species re-reported in later literature based on earlier original publication. Twenty one (17%) of these species were validated by an independent herbarium report. Eighty-four singleton species were reported in the herbarium data (including eight species whose multiple reports originated from plant collections made in the same proximity by the same collector in the same year, since this is considered resulting from one observation). Of these 84, 39 (46%) were validated by a literature report. From the literature, there were 53 singleton species for which there was no information about how the species was used; use reports were available from the herbarium for 6 of these (1 for therapeutic application). From the herbarium, there were 73 singleton species without use record; use reports were available from the literature for 27 of these (27 for therapeutic application, 24 for mode of use, and 25 for plant part used). Considering the 94 shared species, herbarium data added 58 new therapeutic indications, 25 new plant parts used, and 16 new modes of application, a total of 99 new uses when compared to literature.

## Discussion

Our study shows that the modern (1900–2010) herbarium specimens we surveyed comprise a significant source of data. They are information-rich, often describe how the plant is used, and overall, the information they contain appears broadly similar to the information recorded in the literature. We were surprised at how frequently specimens contained information about the specific applications of the plants used; we expected that many specimens might simply report that a species was used medicinally. However, of those specimens citing any medicinal use at all, 69% also reported the therapeutic application of the species, 49% the mode of application, and 61% the plant part used. Overall, the number of specimens with medicinal information is low, however. We show here that just 0.2% of the herbarium specimens we examined for this study contain some information about medicinal application of the species represented by the voucher.

Until critical studies of other herbaria are made, it is not possible to determine whether our findings about the proportion of specimens recording ethnomedicinal data apply more generally. Our survey included all specimens, regardless of whether the species were previously known to have use, yet this proportion is less than the 0.27% reported in von Reis’ studies even after she excluded species already known (von Reis 1962; Von Altschul 1968; Harvard Herbarium) and much less than another study reporting 3% of specimens with information about medicinal use (Shinde and Prakash 2015; Mumbai’s 109-year-old Blatter herbarium). Why this might be is unclear. Few of our herbarium specimens are as old as those in the Blatter herbarium, and we also expect the age profile of the specimens we examine to be younger than the Harvard specimens examined by von Reis. Her survey was carried out in the 1960s; fewer than 2% of the specimens we examined pre-dated 1900 (no pre-1900 specimens included medicinal information), and we do not include specimens collected before 1900. Very early collections were often made by botanists who were trained physicians or made in the context of colonial expansion through exploitation of plant resources and thus were made to record plant use (Schiebinger 2004; Van Andel et al. 2012). Historical herbaria, and the significant national and institutional herbaria they have been bequeathed to and incorporated into, might therefore be richer in ethnobotanical information that the more modern herbaria we surveyed. Yet, we also show that later herbarium specimens in our study are more likely to include medicinal information (Fig. 2), suggesting a renaissance in Brazil in recording plant uses on herbarium labels. This might reflect the exponential growth in the number of individuals and institutions involved in ethnobotanical studies and the recent formal inclusion of ethnobotany in undergraduate and graduate courses in Brazil (Fonseca-Kruel et al. 2005). Outside of Brazil, we might also expect different herbaria, emphasizing specimens from different geographical regions, to include different proportions of specimens with label data reporting medicinal use. This might be because the label-making practices of collectors lodging their specimens in different herbaria differ or because documentation of ethnobotanical uses is more incomplete in some areas (Cámara-Leret et al. 2014a,b).

There are caveats about the quality of medicinal plant use data from herbarium specimens. Unlike publications, which report how the data were collected and from whom, the herbarium labels are silent about how the information was gathered. There are no data about the ethnobotanical indices, such as informant consensus, recording the relative importance of the plants used or the distribution of knowledge in a community (Andrade-Cetto and Heinrich 2011). It is even possible that the information about plant use is drawn from someone from outside of the community, such as a field assistant. Nevertheless, our tests to determine whether the overall content of herbarium labels shows that statistically the same plant parts are used in the same ways for the same therapeutic applications, whether data is sourced from herbarium or published sources. Specifically, the rank order of genera used, plant parts used, and modes of application are not significantly different between sources. This statistical significance of the ranks of parts, modes, and applications might hide more subtle differences between the ways in which ethnobotanists and general collectors report plant use. For example, the use of teas (extracts infused by pouring boiling water over them) is more commonly reported for the herbarium data than in literature reports, where decoctions (extracts concentrated by boiling) are more important. This might reflect a failure of more general collectors to distinguish between teas and decoctions. Similarly, the use of leaves may be underreported in the herbarium labels, since leaves could be considered the “default” plant part, whereas in ethnobotanical literature that reports the plant parts used, use of leaves would be expected to be reported. Similar data content between publications and literature could be due to double-recording, if the authors of the publications have deposited specimens describing the uses they also document in their paper. We, as far as possible, rule out this explanation for shared patterns by showing that the collector names on the specimens were the same as the author names of the publications only 3.6% of the time.

Whereas the genera recorded, and the way plants in general are used, are comparable between herbaria and literature, the geographical distribution of data is less similar. Ritter et al. (2015) reported in their survey of ethnobotanical research in Brazil that the Atlantic Forest and Caatinga biomes are most studied and that the Amazon, Pampa, and Pantanal less frequently so. Our data from literature are not directly comparable, since we record numbers of reports, not articles, and the details of our research strategies differ. Nevertheless, we also find fewer literature reports from the Amazon, Pampa, and Pantanal. Considering data from the herbarium, the same three biomes, Caatinga, Cerrado, and Atlantic Forest, are also best known. However, the proportions of data from each source differ. For the less studied Pantanal, 58% of reports and 62% of the species known are known from the herbarium. Thus, the herbarium goes some way towards meeting the deficit of information from this biome. This is not so for the Pampas, which lacks herbarium reports even more than literature reports (just 16% of reports and 18% of species from herbaria), nor for the Amazon biome (39% of reports and 39% of species from herbaria). For the Amazon, this might be because as Ritter et al. (2015) note, most Amazonian studies have been carried out by workers from outside of Brazil and possibly related to governmental constraints on issuing permits for data collection. If these collections were made before a precedent for depositing Brazilian vouchers in Brazilian herbaria was established, the paucity of Brazilian specimens in our study with data could be because general collectors recording ethnobotany of the Amazon have not deposited their specimens in the herbaria we have surveyed. In general, these herbaria include fewer recent specimens from the Amazon, because of the logistical challenges of conducting fieldwork in remote areas and under challenging conditions, including among communities less likely to speak Portuguese. Although the herbarium data are also few in areas with few literature reports, Fig. 1 shows that data from otherwise unstudied sites are incorporated by including herbarium specimens in any study.

Beyond filling spatial gaps in our knowledge, herbarium data might validate and augment literature reports. We considered that there was a need for validation of species with single reports. There are “legitimate” reasons a species might be cited only once; it might be genuinely rarely used, secret or specialist traditional knowledge or unshared uses (see Cámara-Leret et al. 2014a,b). Alternatively, a single citation might result from misidentification. A second citation validates these reports. Misidentification is widespread in herbaria; it is estimated that more than 50% of tropical specimens are incorrectly named (Goodwin et al. 2015). There is no reason to suppose that species identifications in ethnobotanists’ studies are any more accurate; many identifications in the literature might be wrong. Herbarium specimens might be correctly determined when examined by experts at a later date; for this reason, we might expect them to be more accurate. However, ethnobotanists frequently collect sterile material, whereas botanists almost never do, making it less likely that correct identification would take place as part of curation of herbarium collections. We found medicinal use of 21 of the 128 species known from only one report in the literature that were substantiated from independently collected herbarium specimens. Herbaria are shown here to add information to what is known from the literature. “Augmenting” literature reports in this way could therefore be another important use of herbaria. As we demonstrate, herbaria can add significant data over what is known in the literature by specifying the use of plants.

Studies synthesizing regional or global plant use data may become more common as ethnobotanical research encompasses large scale or meta-analysis (Albuquerque and de Medeiros 2012). Recent studies using datasets compiled from literature have tested whether there are global patterns of plant use between distantly related people (Saslis-Lagoudakis et al. 2012) and determined the relative importance of different drivers of plant selection at national levels (de la Torre et al. 2012; de Medeiros et al. 2013; Saslis-lagoudakis et al. 2014). Ethnopharmacological reviews also depend on the synthesis of data describing the therapeutic applications and modes of use of a taxon before interpreting use in the light of chemistry and pharmacological activity (Fernandes and Banu 2012; Seebaluck et al. 2015; Wang et al. 2013). Reviews can highlight knowledge gaps and prioritize communities and areas for field research (Uprety et al. 2012); nevertheless, the scope and reach of many meta-analyses in ethnobotany demand more data than can be delivered by directed fieldwork programs. The availability of data and its quality poses a significant barrier to further study (de Medeiros et al. 2013). This deficit of information, for certain types of study, might be overcome by using herbaria.

Herbaria are irreplaceable resources; the long-term capital investment in specimen acquisition and curation that they represent is increasingly rewarded across many fields (Funk 2003). Our study, by explicitly quantifying ethnobotanical data from herbaria and literature, demonstrates that herbaria contain valuable information that can both supplement and complement literature reports. Visits to herbaria to check specimens for associated data would be extremely time consuming; our study was facilitated by fast, easy access to data from online herbaria. The ease in which data are accessed is set to increase as digitization projects progress (Haston et al. 2012), generating more and more accurate data. Ethnobotanical equivalents of the Darwin Core Biodiversity Data Standards (Wieczorek et al. 2012) might be envisaged, to maximize the value of digitization efforts. We anticipate a wider role for herbarium data in the future.

## Electronic supplementary material

Below is the link to the electronic supplementary material.

ESM 1 (DOCX 236 kb)
